# Cloned Defective Interfering Influenza Virus Protects Ferrets from Pandemic 2009 Influenza A Virus and Allows Protective Immunity to Be Established

**DOI:** 10.1371/journal.pone.0049394

**Published:** 2012-12-12

**Authors:** Nigel J. Dimmock, Brian K. Dove, Paul D. Scott, Bo Meng, Irene Taylor, Linda Cheung, Bassam Hallis, Anthony C. Marriott, Miles W. Carroll, Andrew J. Easton

**Affiliations:** 1 School of Life Sciences, University of Warwick, Coventry, United Kingdom; 2 Health Protection Agency, Porton Down, Salisbury, United Kingdom; Instituto Butantan, Brazil

## Abstract

Influenza A viruses are a major cause of morbidity and mortality in the human population, causing epidemics in the winter, and occasional worldwide pandemics. In addition there are periodic outbreaks in domestic poultry, horses, pigs, dogs, and cats. Infections of domestic birds can be fatal for the birds and their human contacts. Control in man operates through vaccines and antivirals, but both have their limitations. In the search for an alternative treatment we have focussed on defective interfering (DI) influenza A virus. Such a DI virus is superficially indistinguishable from a normal virus but has a large deletion in one of the eight RNAs that make up the viral genome. Antiviral activity resides in the deleted RNA. We have cloned one such highly active DI RNA derived from segment 1 (244 DI virus) and shown earlier that intranasal administration protects mice from lethal disease caused by a number of different influenza A viruses. A more cogent model of human influenza is the ferret. Here we found that intranasal treatment with a single dose of 2 or 0.2 µg 244 RNA delivered as A/PR/8/34 virus particles protected ferrets from disease caused by pandemic virus A/California/04/09 (A/Cal; H1N1). Specifically, 244 DI virus significantly reduced fever, weight loss, respiratory symptoms, and infectious load. 244 DI RNA, the active principle, was amplified in nasal washes following infection with A/Cal, consistent with its amelioration of clinical disease. Animals that were treated with 244 DI RNA cleared infectious and DI viruses without delay. Despite the attenuation of infection and disease by DI virus, ferrets formed high levels of A/Cal-specific serum haemagglutination-inhibiting antibodies and were solidly immune to rechallenge with A/Cal. Together with earlier data from mouse studies, we conclude that 244 DI virus is a highly effective antiviral with activity potentially against all influenza A subtypes.

## Introduction

Human influenza is a debilitating respiratory disease which arises from seasonal winter outbreaks and catastrophic world-wide pandemics [1]–[3]. Measures to combat influenza include vaccines [4]–[6] and antivirals [7]–[9]. Both have their strengths and weaknesses. Current vaccines are highly specific and to be effective have to be closely matched to the prevailing virus and immunity takes 1–3 weeks to reach maximum efficacy. People whose immune system is compromised (including the elderly) may not make a fully protective immune response or their immunity may wane too quickly to be effective [10], [11]. The antivirals oseltamivir and zanamivir protect against all influenza A and B strains and can be used prophylactically or therapeutically. Oseltamivir and zanamivir are administered twice daily, and are most effective when taken before or soon after infection. However, oseltamivir-resistant mutants were already wide-spread in seasonal H1N1 virus prior to 2009 [12], [13], and are now appearing in the 2009 pandemic virus [14]–[16]. More counter-measures, especially those that are broad-spectrum and not subject to virus resistance are urgently needed.

Our approach is to use defective-interfering (DI) virus [17], which is produced by nearly all viruses during their replication process, as an antiviral *in vivo*. DI viruses are widely known to have powerful antiviral (interfering) activity in cell culture but, with a few notable exceptions, there is little indication that this activity translates into conferring protection from disease in animal models [18]. One of the first accounts, more than 60 years ago, refers to what is now known as DI influenza virus [19], [20]. A DI influenza virus is defective as it has a large deletion in the central region of one of the 8 viral RNA segments that constitute the infectious genome. This is a DI RNA. Not all defective RNAs are interfering, hence interference is a particular property of a certain class of defective RNA. Influenza DI particles are indistinguishable from normal infectious particles except in that they contain at least one genomic segment that has undergone a massive deletion. Any one of the 8 genomic segments can give rise to a DI RNA, although DI RNAs arise most frequently from segments 1, 2 or 3. The situation is made more complex as the position of the central deletion in any one segment can vary considerably, so giving rise to many different DI RNA sequences. Natural populations of DI influenza virus can contain greater than 50 different defective RNA sequences [21], [22], and initially it was impossible to produce a defined DI virus preparation that could be quality controlled. We solved this problem by using cloning techniques to isolate single, naturally occurring DI RNAs of known sequence [22]. One of these DI RNAs, originating from segment 1 (called 244), was incorporated by reverse genetics into A/PR/8/34 influenza virus to form the DI virus 244/PR8. This DI virus is highly effective in protecting against clinical disease caused by a lethal influenza challenge in a mouse model [23]. In mice a single intranasal dose administered before or at the same time as the infectious virus completely suppresses clinical disease symptoms caused by H1N1, H2N2, H3N2 or H3N8 viruses also given intranasally. Post-infection therapy is also effective. 244/PR8 is not toxic and generates no adverse clinical effects, and the administered preparation is not infectious. Different strains of mice and elderly mice are well protected [24]–[26]. Infection of mice with severe-combined immunodeficiency (SCID) shows that the adaptive immune response is not required for protection but is needed to clear infection [24]. Interferon is made locally in the respiratory tract in response to DI virus but is not required for protection from influenza A viruses. However, interferon affords protection from heterologous respiratory viruses such as influenza B and a paramyxovirus, pneumonia virus of mice [26], [27]. On this basis DI virus offers a potentially attractive new addition to the armoury of anti-influenza treatments.

A DI influenza virus is not capable of independent replication as it contains at least one defective RNA segment. However, when an infectious virus particle enters a cell which a DI virus has already invaded, it replicates not only its own full length RNA segments but also the DI RNA. Little is known of the details of the mechanism by which an influenza DI virus achieves its antiviral effects, but it is characteristic of all DI viruses that defective particles come to predominate over infectious virus in a cell culture [28]. All influenza DI RNAs retain the common replication and packaging signals that are located at the termini of each RNA segment [8], [23] suggesting that the DI genome is recognised by and subverts the viral replication machinery so that DI RNAs are made in preference to full-length genomic RNAs. In addition there is probably preferential packing of the DI genome with the cell producing more non-infectious DI virus than infectious virus particles [29]. It is envisaged that protection *in vivo* against influenza A viruses operates through decreasing the infectious virus load, while increasing the production of DI RNA and DI virus. This significantly reduces or obviates clinical disease, and gives the adaptive immune system time to become activated and clear the infection. Because the majority of progeny virus (both DI and infectious virus) is packaged by the proteins of the infecting virus, a solid adaptive immunity is generated that protects mice from further infection by the same strain of virus [23], [25].

Although we have extensive proof of concept in mice, protection of ferrets is the accepted pre-clinical acid test for influenza vaccines and antivirals. Ferrets are highly sensitive to infection by human influenza viruses [30], [31], and mount a disease that closely resembles that in humans [32]–[36]. Ferrets have been used in many aspects of influenza biology including the study of recent human [37], [38] and avian influenza viruses [39]–[43], airborne transmission [36], [37], [44], the evaluation of vaccines [6], [14], [19], testing antivirals [45], [46], and the interaction with bacteria in the respiratory tract [47]. We have evaluated the ability of a single intranasal dose of cloned DI virus (containing as little as 0.2 µg of antiviral DI RNA) to protect ferrets from the recent 2009 pandemic influenza A virus (A/California/04/09, H1N1). We report that all clinical signs of disease were significantly reduced and that animals mounted a solid virus-specific antibody response and became immune to reinfection with A/California/04/09.

## Results

### Influenza in ferrets caused by the pandemic influenza virus A/California/04/09 (H1N1) (A/Cal)

Typically ferrets infected with A/Cal (10^2^ TCID_50_) show a peak of virus infectivity in nasal washes on days 2 and 3, which then declines and is undetectable by day 8. The peak of infectivity is followed one day later by a fever spike (>40°C) and a significant but transient weight loss (day 3). There is an increase in the number of small round cells in nasal washes that commences on day 2 and peaks on day 3. This declines slowly and is still above baseline on day 14. The peak of infection is accompanied by pronounced nasal discharge and sneezing. However, there is no significant loss of appetite, loss of activity, or diarrhoea (data not shown).

### 244 DI virus reduces fever in A/Cal-infected ferrets

**Figure 1:**
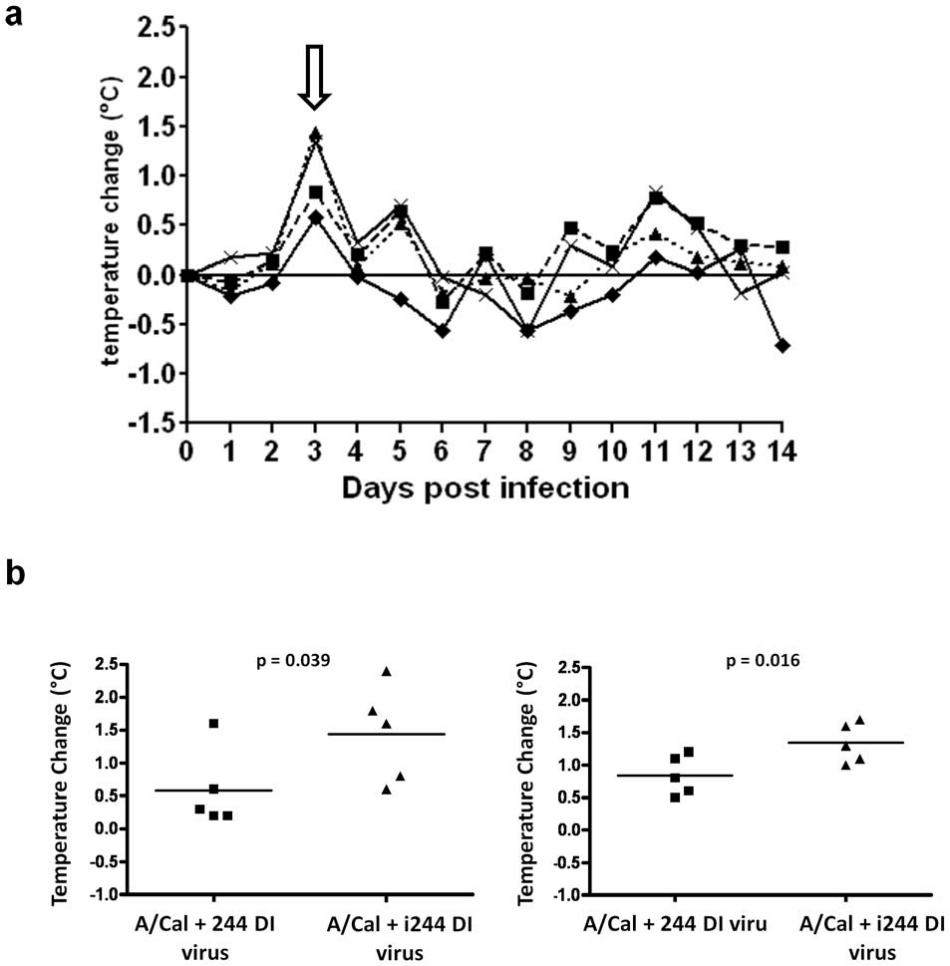
Change in rectal temperatures in ferrets infected with A/California/04/09 (H1N1) and treated with 244 DI virus simultaneously on day 0. Ferrets were treated with A/Cal+300 µg 244 DI virus (⧫); A/Cal+30 µg 244 DI virus (▪); A/Cal+300 µg inactivated 244 DI virus (▴); A/Cal+30 µg inactivated 244 DI virus (x). In (a) the mean changes in temperatures of each group (n = 5) are expressed as the difference to the group average temperature on day 0. Animals were anaesthetised and rectal temperatures taken prior to any other procedure. The arrow denoted the fever spike on day 3 post infection/treatment. In (b) the statistical significance of temperature change differences on day 3 after infection/treatment with 300 µg (left-hand panel) or 30 µg (right-hand panel) 244 DI virus or inactivated (i) 244 DI virus were determined using a one tailed unpaired t-test. The mean temperature change seen in ferrets treated with 300 µg 244 DI was 0.58°C (SD ±0.19°C) and in those treated with 300 µg inactivated 244 DI virus was 1.44°C (SD ±0.74°C). The mean temperature change seen in ferrets treated with 30 µg 244 DI was 0.84°C (SD ±0.30°C) and in those treated with 30 µg inactivated 244 DI virus was 1.34°C (SD ±0.30°C).

Groups of 5 ferrets were infected intranasally with A/Cal (10^2^ TCID_50_). Animals were treated at the same time with an intranasal dose of active 244 DI virus containing approximately 2 µg or 0.2 µg of active agent (244 RNA) in 300 or 30 µg respectively of virus protein or with UV-inactivated 244 DI virus (300 or 30 µg). Figure 1A shows that the control infected ferrets treated with either 300 or 30 µg of inactivated 244 DI virus displayed a fever spike on day 3 post infection (pi) which is a clear sign of disease. Infected animals treated with active 244 DI virus had significantly lower temperatures on day 3 compared to the control group treated with inactivated 244 DI virus. The mean group temperature rises for infected animals treated with 300 µg 244 DI virus or inactivated 244 DI virus respectively were 0.58°C and 1.44°C (Student's t-test p = 0.039). Similarly, ferrets treated with 30 µg of 244 DI or inactivated 244 DI experienced mean temperature rises of 0.84°C and 1.34°C (p = 0.016) (Fig. 1B). Control non-infected animals treated with 300 µg of 244 DI virus or saline had no fever peak (data not shown).

Temperature changes were also recorded by subcutaneously implanted transponders. These confirmed a fever peak on day 3 in infected animals given control inactivated 244 DI virus. Animals treated with 300 µg of active 244 DI virus had significantly less fever than animals treated with the same amount of inactivated 244 DI virus (0.16°C increase compared with a 1.2°C increase, p = 0.025) while animals treated with the lower dose of 30 µg 244 DI virus also had significantly less fever than animals treated with the same amount of inactivated 244 DI virus (0.02°C for 244 DI virus and 0.84°C for inactivated 244 DI virus, p = 0.009) (data not shown).

The data show that active 244 DI virus significantly reduces the fever response in ferrets infected with A/Cal. The 30 and 300 µg doses of 244 DI virus gave similar protection.

### 244 DI virus reduces weight loss in A/Cal-infected ferrets

**Figure 2:**
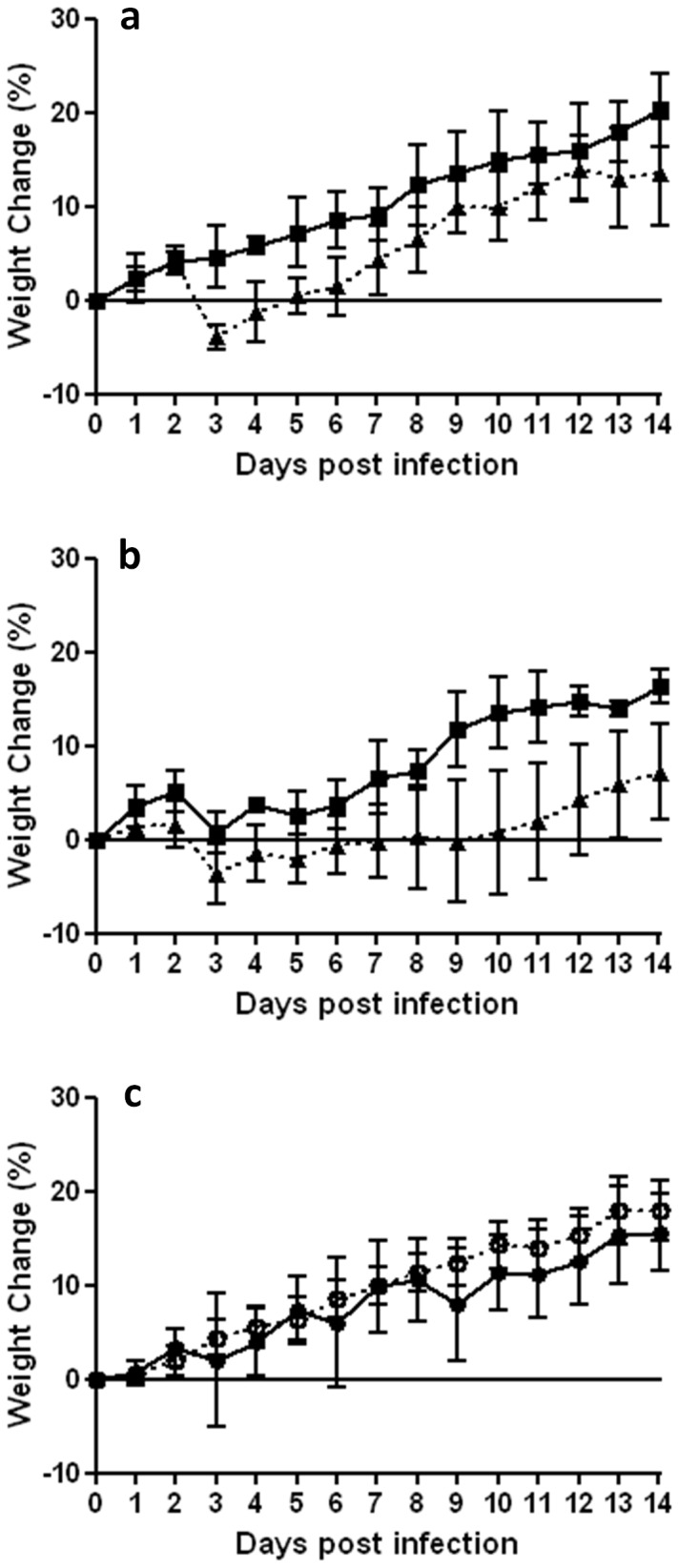
Changes in weight of ferrets over the course of infection with A/Cal. Shown is the mean group body weight changes in A/Cal influenza virus-infected ferrets treated with (a) 300 µg 244 DI virus (▪) or inactivated 244 DI virus (▴), (b) 30 µg 244 DI virus (▪) or inactivated 244 DI virus (▴). (c) Shows the weight changes in ferrets inoculated with saline (○) or treated with 300 µg of active 244 DI virus (•). Data are expressed as a percentage change compared to the group average weight at day 0. The statistical significance of body weight changes on any one day was determined by a one tailed unpaired t-test and is indicated by an asterisk (p≤0.05).

A/Cal-infected ferrets treated with 300 µg 244 DI virus showed a smooth increase in weight over the period of observation (Fig. 2A). In contrast, infected animals treated with inactivated 244 DI virus showed a pronounced and highly significant weight loss on day 3, falling to below their initial mean group weight. These animals then resumed weight gain but from days 3–8 their weights were significantly lower than the 244 DI virus-treated group, and from days 9–14 were all still lower than the 244 DI virus-treated group at the end of the experiment, although not significantly so. Animals treated with 30 µg 244 DI virus showed a small weight loss on day 3 but weights were still significantly greater than the control group treated with an equivalent amount of inactivated 244 DI virus from days 3–14 (Fig. 2B). Ferrets treated with 300 µg active 244 DI virus (but not infected with A/Cal) gained weight steadily at a very similar trajectory to those given saline, indicating that the higher dose of 244 DI virus had no deleterious effect of weight gain (Fig. 2C).

### 244 DI virus reduces respiratory disease (sneezing and nasal discharge) in A/Cal-infected ferrets

**Figure 3:**
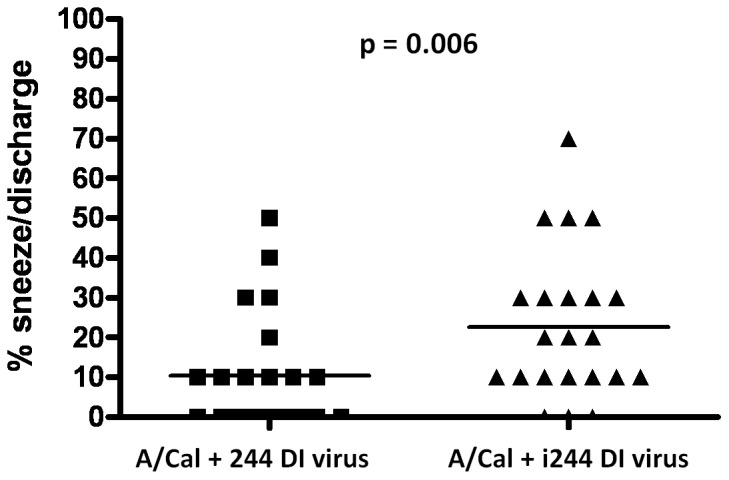
Analysis of combined sneezing and nasal discharge data in A/Cal infected ferrets treated with 300 µg of active 244 DI virus (▪) or 300 µg of inactivated (i) 244 DI virus (▴). The horizontal line indicates the mean: 244 DI virus mean was 10.45%; inactivated 244 DI virus mean was 22.73%. The values are the occurrence of sneezing and nasal discharge events in each group over the 14 days of observation. The scores were assigned 10% for each individual event such that all 5 ferrets were positive for both sneezing and nasal discharge would be scored as 100%; a.m. and p.m. assessments are separate. Zero was only scored when the other group was positive. The p value was determined using a one tailed Mann-Whitney U test.

Sneezing and nasal discharge are typical clinical signs of influenza, and ferrets were monitored twice daily for changes in these parameters. Data for combined sneezing and nasal discharge observations are shown in Figure 3, where the reductions effected by 244 DI virus were highly significant (one tailed Mann-Whitney U test, p = 0.006). 300 µg 244 DI virus reduced sneezing and nasal discharge calculated in a variety of combinations, with all being statistically significant (p≤0.05). 30 µg 244 DI virus had lower efficacy, but significantly reduced nasal discharge (Table S1).

### 244 DI reduces A/Cal infectivity in ferret nasal washes

**Figure 4:**
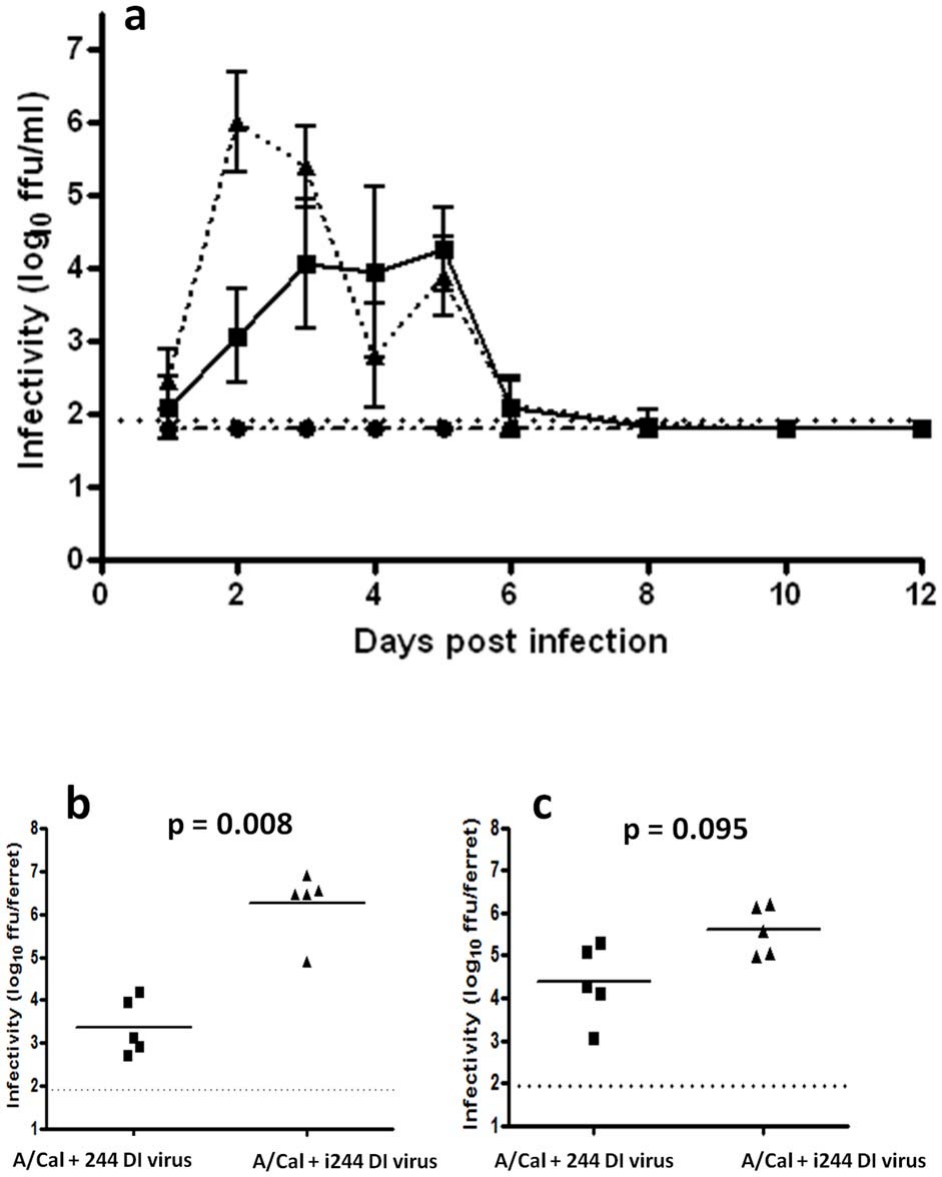
Summary of A/Cal infectivity in nasal washes. Panel (a) shows ferrets infected with A/Cal on day 0 and treated with 300 µg 244 DI virus (▪) or infected and treated with 300 µg inactivated 244 DI virus (▴); another group was not infected but treated with 300 µg of active 244 DI virus (•). A standard preparation of A/Cal virus was used to normalise titrations carried out on different days. These varied by less than 4-fold. The dotted line is the limit of sensitivity of the assay (1.92 log_10_ FFU/ml). Significant reduction in infectivity (by a two-tailed Mann-Whitney U test) in ferrets treated with 244 DI virus is denoted by **. Panels (b) and (c) show details of the statistical analysis on day 2 and 3, respectively.

Nasal washes were taken daily from ferrets and assayed for infectious A/Cal and for 244 DI RNA (see next section). Figure 4A shows that low amounts of infectivity appeared in control ferrets treated with inactivated 244 DI virus in day 1, peaked on day 2, and then began to decline from day 3. In animals treated with 300 µg 244 DI virus infectious virus was also first detected on day 1, but peaked over days 3–5, being both delayed and reduced. The reduction of infectivity by 244 DI virus on day 2 (764-fold) was highly significant (p = 0.008); the reduction on day 3 was 17-fold and was also significant (p = 0.095), both using a two-tailed Mann-Whitney U test (Fig. 4B, 4C). Infectivity levels in DI virus-treated animals were maintained until day 5, and were undetectable on day 8, showing that 244 DI virus did not prolong infection (Table S2). The dynamics of infectious virus in animals treated with a 10-fold lower dose of 244 DI virus (30 µg) were very similar to those observed with the higher DI virus dose.

### 244 DI RNA is hugely amplified in nasal washes of ferrets infected with A/Cal

**Figure 5:**
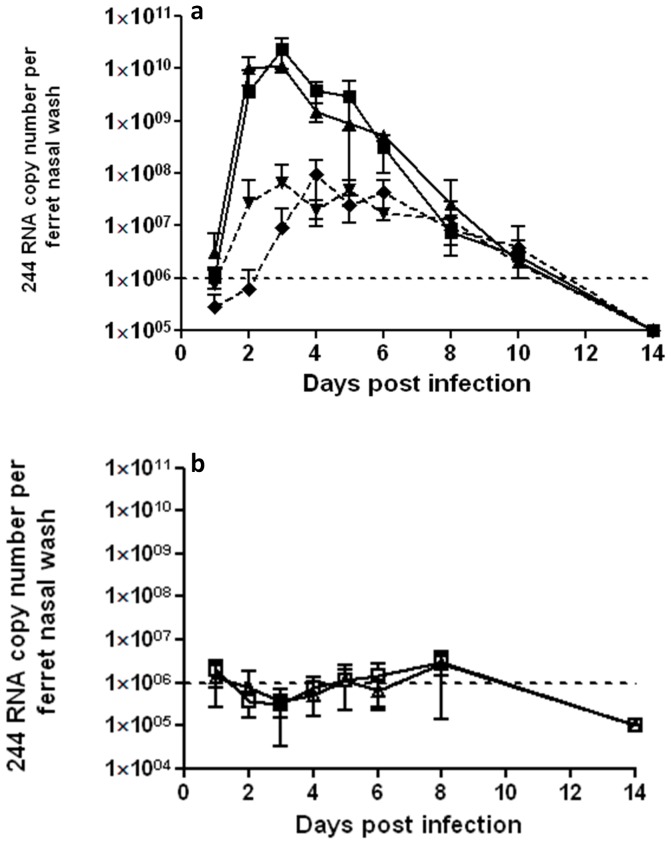
244 DI virus (244) RNA is amplified in nasal washes by A/Cal. Ferrets were infected with A/Cal on day 0 and treated with 244 DI virus or inactivated 244 DI virus. Levels of 244 DI RNA were determined by quantitative RT-PCR. Mean 244 RNA copy numbers for each ferret group (n = 5) are plotted. Panel (a) shows ferrets that were infected with A/Cal influenza virus and treated with 300 µg 244 DI virus (▪), or 30 µg 244 DI virus (▴), or 300 µg (i) inactivated 244 DI virus (▾), or 30 µg (i) inactivated 244 DI virus (⧫). Panel (b) shows non-infected ferrets that were given 300 µg 244 DI virus (□), or diluent (▵). The dotted line shows the limit of detection. Figure S1 gives details for individual animals.

244 RNA is the antiviral active principle responsible for protection from influenza A virus infection [23]. To determine how this interaction proceeded in the ferret, nasal washes were assayed for the presence of DI virus-specific 244 RNA by quantitative RT-PCR. Figure 5 shows that 244 DI RNA was below detectable levels in animals treated with 244 DI virus at 1 day after treatment. However, by day 2, 244 DI virus RNA levels had increased by ≥1000 to 10,000-fold, showing that it was being replicated by A/Cal. Amounts of DI RNA in the individual ferrets within a group were highly reproducible (Figure S1). The small amount of 244 DI RNA in the groups given inactivated DI virus and A/Cal is the result of incomplete destruction of DI RNA. Levels of DI RNA declined and were undetectable by day 10 after infection. There was no discernible difference in the 244 DI RNA dynamics in ferrets treated with 300 µg or 30 µg of active 244 DI virus. These data demonstrate for the first time in ferrets the ability of the 244 DI RNA to be amplified by the agent that it is acting against – in this case A/Cal influenza virus. This observation is fully consistent with data arising from the mouse model [23], [24].

### Other clinical parameters

Ferrets were also monitored over 21 days post infection for diarrhoea (1 instance reported in each of infected 2 ferrets), change (loss) of appetite (none recorded), and changes in activity/behaviour (7 instances recorded in 4 different groups). None was considered significant. The number of cells in nasal washes increased by approximately 50-fold in all A/Cal-infected animals compared with the saline-treated group, although 244 DI virus on its own did not stimulate the cell content of nasal washes, and did not differ from the group treated with saline (data not shown). It seems therefore that the increase in cells in nasal washes is a response to infectious influenza virus. In addition serum HI titres specific to A/California/04/09 (H1N1) taken at 14 days after infection were unaffected by the treatment with 244 DI virus, with titres in all A/Cal-infected groups rising from <1/40 to >1/8000 (data not shown).

### Ferrets protected from A/Cal with DI virus are immune to reinfection with A/Cal

**Figure 6:**
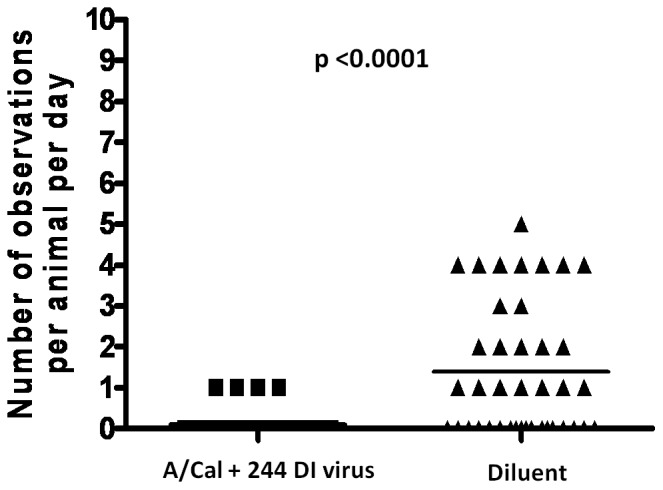
Statistical analysis of summed clinical signs for each day in ferrets re-challenged with A/Cal. The group that previously experienced A/Cal+300 ug 244 DI virus (▪) is compared with the group that previously experienced only saline (▴). The p value was determined using a one tailed Mann-Whitney U test.

We have previously shown that mice protected by 244 DI virus were solidly immune to rechallenge with the same virus [23]. This allayed concerns that, following treatment with 244 DI virus, the challenge infection had been attenuated and might result in a weaker adaptive immune response. To determine the situation in ferrets, the groups initially receiving A/Cal+300 µg active 244 DI virus, A/Cal+300 µg inactivated 244 DI virus, and a saline inoculum were challenged with A/Cal at 21 days after the initial A/Cal infection. A dose of (10^6^ TCID_50_) was chosen as high doses of influenza virus overcome the protection mediated by DI virus in mice (unpublished data). Thus any protection observed could be attributed to adaptive immunity. Naïve animals showed a peak mean rise in rectal temperature (0.96°C) on day 2, while the temperature of other previously infected groups increased by ≤0.2°C. This difference was statistically significant (p≤0.03). These animals were protected regardless of treatment with DI virus (Figure S2). Temperature recording by transponder chip gave a similar result with a temperature spike only in naïve animals (of 0.66°C compared with ≤0.18°C for the other groups) (data not shown). Only naïve animals experienced significant weight loss following challenge, also on day 2. In particular, A/Cal-infected ferrets that had previously been treated with 244 DI virus were highly significantly better at gaining weight over days 2–7 than those treated with inactivated 244 DI virus (Figure S3). Table S3 summarizes particulars of sneezing, nasal discharge, activity loss and appetite loss in individual ferrets. Naïve animals were positive for all clinical signs on at least 2 observation days following challenge. Most, if not all, animals displayed one or more clinical sign during the observation periods. In contrast, groups that had previously been infected with A/Cal showed only very occasional signs of sneezing, and no sign of disease. The reduction in clinical signs in non-naïve animals compared to the naïve control was highly significant (p<0.0001) (Fig. 6). In conclusion, ferrets that had been protected from influenza by treatment with 244 DI virus were able to mount an immune response that protected them from subsequent challenge with the same virus in the same way that was seen with control infected animals. This finding is entirely consistent with the high serum HI antibody titres found in all A/Cal-infected ferret groups, as reported above.

## Discussion

The ferret model of influenza is viewed as the closest to the human disease, and is widely used to evaluate vaccines and other anti-influenza measures. In this study we infected ferrets with an isolate from the latest influenza pandemic (A/California/04/2009). The possibility that DI virus might have a moderating effect on influenza has long intrigued virologists but the only data relevant to the ferret model is our preliminary study in which animals were protected using an intranasal dose of a DI virus preparation which contained an unspecified number of DI RNAs [48]. Here we have investigated the protection afforded by a fully defined, cloned DI RNA delivered in an influenza virus particle. The delivery vehicle was carefully selected to recognise both the α2,6 and α2,3 sialic acid receptors recognised by influenza A viruses [49], so that 244 DI RNA is delivered to cells that can potentially be infected by an incoming influenza virus. This is important as when we treated ferrets with 244 DI RNA in a strain of PR8 that recognises predominantly α2,3 sialic acid receptors there was no amelioration of infection by infectious virus that recognised predominantly α2,6 sialic acid receptors. In that study the DI RNA was eventually amplified by the challenge virus A/Sydney/5/97 (H3N2), but presumably too late to be of any clinical benefit (unpublished data).

The data above (summarized in Table 1) show clearly that both doses of 244 DI RNA (300 or 30 µg of DI virus containing 2 or 0.2 µg of 244 DI RNA, respectively) significantly ameliorated clinical disease caused by A/Cal infection. The higher dose resulted in a reduction in every clinical parameter tested (fever, weight loss, sneezing and nasal discharge, and infectious load), which was concomitant with the enhancement of 244 RNA in nasal washes. DI RNA was undetectable by a sensitive quantitative RT-PCR at 1 day post treatment, but was amplified on infection, and appeared in nasal washes at the same time as infectious virus. The overall reduction in symptoms was similar to that observed with oseltamivir treatment of ferrets infected with the same virus, where the animals were treated within 2 hours of infection and then twice daily for 5 days [50]. The present study used only a single very low dose of DI RNA (2 or 0.2 µg), whereas most antivirals are used in milligram amounts with multiple doses. It is also relevant to the success of the treatment that infectious A/Cal was not detected beyond 8 days post infection, and that the DI RNA was not detected beyond 10 days by quantitative RT-PCR: the presence of amplified active DI RNA made no difference to the dynamics of clearance. 244 DI RNA amplified by A/Cal would be expected to be packaged as a DI virus particle with proteins, including the surface haemagglutinin and neuraminidase, synthesised by A/Cal. Further, it is likely that this DI 244/Cal virus transmitted its 244 DI RNA to other cells in the respiratory tract where it protected them from infection. In principle, such a DI 244/Cal virus may also be transmitted in airborne droplets/aerosols of nasal secretions, along with infectious virus, to other individuals. It will be of interest to determine how DI virus might alter the dynamics of influenza transmission and infection.

**Table 1:** Summary of 244 DI virus-mediated protection of ferrets from influenza caused by A/Cal.

Benefit of treatment	244 DI virus	
	300 µg	30 µg
**Reduction in fever by rectal temperature recording**	Yes*	Yes
**Reduction in fever by chip temperature recording**	Yes	Yes
**Reduction in loss of body mass**	Yes	Yes
**Reduction in sneezing**	Yes	No
**Reduction in nasal discharge**	Yes	Yes
**Reduction in combined sneezing & nasal discharge**	Yes	No
**Reduction of A/Cal infectivity in nasal washes**	Yes	Yes
**Increase in 244 DI RNA in nasal washes**	Yes	Yes
**Immunity to rechallenge with A/Cal**	Yes	ND

* **Statistically significant at ≥95% confidence limits; data presented elsewhere in this report.**

**ND, not done.**

One important proviso of treating a virus infection is that the immune response is not so weakened that the cognate adaptive immune response is compromised. Accordingly, we tested immunity measuring the amount of virus-specific serum antibody by HI and by challenging animals with a second dose of A/Cal. Despite the amelioration of the A/Cal-mediated clinical disease by treatment with DI virus we found that there were similar levels of HI antibody in all infected groups. Animals re-challenged with A/Cal at 21 days were solidly protected from disease. This protection was most likely mediated by serum HI antibody, which is known to be protective, and other adaptive immune responses rather than by residual DI RNA, as DI RNA could not be detected in nasal wash or tissues (data not shown) at 14 days after the first infection. Innate immune responses stimulated by the original infection are probably not a factor in protection at the time of rechallenge, as most of these responses which rose rapidly in ferrets in concert with the symptoms of influenza, had declined significantly by 6 days post infection [51].

The antiviral activity resulting from intranasal administration of 244 DI virus in ferrets has a number of unique features. Protection requires only a single dose, a very small amount (0.2 µg) of DI 244 RNA, and delivery to cells by 10^4^ HAU of an influenza A virus (approximately 30 µg) that recognises α2,6 and α2,3 sialic acid receptors [49]. DI RNA is amplified only if cells are infected by an influenza A virus, otherwise the DI RNA degrades. In mice protection mediated by a single critical dose of 244 DI virus is lost 1–2 weeks after treatment (unpublished data). Treatment with DI RNA does not affect clearance of infectious influenza virus, and the DI RNA itself rapidly disappears from nasal washes. In addition, in mice DI virus stimulates sufficient interferon type I to protect against respiratory disease caused by non-influenza virus although this is not needed for protection from influenza A viruses [26], [27]. The development of resistance to 244 DI virus has not been detected. For this to occur, the polymerase, which is encoded by infectious virus and is used by all 8 full-length segments, would have to acquire amino acid changes such that it no longer recognised the DI RNA while at the same time acquiring compensating mutations in all 8 full-length segments simultaneously (unpublished data). Given a mutation rate of 10^−5^, the chance of this occurring is approximately 10^−40^, hence this is unlikely to arise. Finally, A/Cal-infected animals that are protected by DI RNA mount a normal HI antibody response and are solidly immune to rechallenge with the same virus. The treatment demonstrated above could be considered directly relevant to treatment of humans who had just been or were about to be exposed to infection. We need in due course to determine the limitations of treatment or after before infection, and if these mirror the data we have already produced in mice [23]. In summary, data here underline the strong antiviral capacity of 244 DI virus and demonstrate how it could be used as a novel antiviral in a clinical context.

## Materials and Methods

### Ethics statement

The experimental animal work described here has been scrutinised and approved by the Ethical Review Committee of the Health Protection Agency (Porton), as required by the UK Home Office Animals (Scientific Procedures) Act, 1986. All animal work was conducted according to UK Home Office legislation for animal experimentation. The premises in which the work was conducted are approved under Home Office Certificate of Designation PCD70/1707.

### Production of cloned 244 DI virus by reverse genetics

244 DI RNA was originally generated spontaneously from segment 1 during the transfection of 293T cells with plasmids required to make infectious influenza A/PR/8/34 [52]. Its sequence has been published [23], [52]. The original 244/PR8 DI virus protected mice from lethal influenza but ferrets were not protected effectively (unpublished data). This related to the receptor specificity of the HA as α2,3-linked sialyl receptor sequences are predominant in the mouse respiratory system, and α2,6-linked sialyl receptor sequences predominant in the ferret respiratory system [53]–[55]. Thus to make the DI virus for the current study we cloned and used the HA and NA proteins from a different substrain of PR8 (A/PR8(Warwick), which binds to both α2,6- and α2,3-linked sialyl receptors [49]. An uncloned DI A/PR8(Warwick) had earlier successfully protected ferrets from an H3N2 virus [48]. The new recombinant virus bound as expected to α2,6- and α2,3-linked sialic acids in a surface plasmon resonance assay (data not shown). Plasmids were transfected into 293T cells and, after 24 h, these were trypsinized, mixed with MDCK cells and re-plated. Resulting virus was passaged once in embryonated chicken's eggs, and the resulting mixture of 244/PR8 DI virus and infectious helper PR8 virus was purified away from extraneous contaminating material by differential centrifugation through sucrose. Stocks were resuspended in PBS containing 0.1% w/v bovine serum albumin, standardized by haemagglutination titration, and stored in liquid nitrogen. Protection activity in mice was comparable to that shown earlier [23]. DI virus was UV-irradiated to remove helper virus infectivity with a short burst (50 seconds) of UV irradiation at 253.7 nm (0.64 mW/cm^2^) (‘active DI virus’ [23]). Longer UV irradiation (8 minutes) inactivates protecting activity for mice although it does not destroy all DI RNA (‘inactivated DI virus’). It does not affect haemagglutinin or neuraminidase activities, and so controls for any immune system-stimulating or receptor-blocking effects of 244 DI virus particles.

### Challenge virus preparation

Pandemic 2009 influenza H1N1 A/California/04/2009 (A/Cal; from CDC Atlanta, GA) was propagated in Madin-Darby canine kidney (MDCK) cells (Health Protection Agency Culture Collection) and titrated by limiting dilution in the same cell type to give a 50% tissue culture infectious dose (TCID_50_).

### Ferret studies

All ferret experimental work was conducted according to UK Home Office legislation for animal experimentation and was approved by the local ethical committee. Thirty male ferrets (*Mustela putorius furo*), 3–4 months of age, were obtained from Highgate Farm, UK. All ferrets were confirmed to be seronegative for antibodies to H1N1 influenza as determined by haemagglutination-inhibition. Ferrets were separated into 6 groups, each comprising 5 animals, and a unique identifier body temperature transponder (idENTICHIP, Bio-Thermo) was inserted subcutaneously into the scruff of each ferret. Ferrets were sedated by intramuscular injection of ketamine (17.9 mg/kg) and xylazine (3.6 mg/kg) then each ferret was intranasally inoculated with 500 µl (250 µl per nare) of challenge solution. Groups of ferrets (n = 5) were infected intranasally with 100 TCID_50_ of the pandemic 2009 influenza virus A/California/04/09 (H1N1) (A/Cal). The DI virus test groups were treated intranasally with a mixture of A/Cal and active 244 DI virus containing approximately 2 µg 244 RNA in 300 of virus protein, or with A/Cal and 0.2 µg 244 RNA in 30 µg of virus protein. Control groups received virus and UV-inactivated 244 DI virus equivalent to the 2 µg RNA dose, virus and UV-inactivated 244 DI virus equivalent to the 0.2 µg RNA dose, or saline. Each inoculum was prepared on the day of challenge and the titre of the virus was subsequently reconfirmed in MDCK cells.

The rectal temperatures of all ferrets were measured daily. Ferrets were monitored twice-daily post-challenge throughout the course of the study for clinical signs indicative of influenza infection (lack of activity, sneezing, nasal discharge, lack of appetite, weight loss and pyrexia). Each animal was monitored for up to 5 minutes by trained animal technicians. Staff were aware of which animals were infected and any treatment received. Clinical signs were scored using a simple matrix depending on severity. Loss of activity was scored; 0 for normal activity levels, 1 for reduced activity and 2 if inactive. Nasal discharge was scored 0 if no nasal discharge was present and 1 if nasal discharge was present. Sneezing was scored; 0 if no sneezing and 1 if ferrets were sneezing. Loss of appetite was scored; 0 for no appetite loss and 1 for loss of appetite. Nasal washes were collected from each ferret following sedation (as above) at days 1–6 and then at days 8, 10 12 and 14 post-challenge. For each nasal wash, 2 ml of PBS were instilled by small multiple volumes into each nasal cavity with expectorate collected into a beaker.

At 14 days post-challenge, ferrets in groups 2, 4 and 5 were sacrificed. Monitoring of ferrets in groups 1, 3 and 6 continued twice daily for days 15–20 post-challenge. At day 21 post-challenge ferrets were sedated as before and inoculated intranasally with 10^6^ TCID_50_ A/Cal in a 500 µl inoculum. Ferrets were monitored for a further seven days twice daily for clinical signs of influenza infection. At 28 days post the first challenge all ferrets in groups 1, 3, 5 and 7 were sacrificed, and blood and tissue samples taken as before.

### PCR assays

RNA was extracted from nasal washes with QIAamp mini RNA kit (Qiagen) and quantitative real time PCR performed to quantitate virion-sense RNA using an ABI prism 7000 [24]. We used the primers and probe: 244 1F (5′ CTCTTTGCCCAGAATGAGGAAT 3′), 244 1R (5′ CATAATCAAGAAGTACACATCAGGAAGAC 3′) and probe (5′ FAM-CCCTCAGTCTTCTCC 3′). Primers were synthesized by Invitrogen, and the probes by ABI. Reverse transcriptase reactions (10 µl) were performed using 6 µl extracted RNA, RevertAid reverse transcriptase and random hexamer (Fermentas) were used according to the manufacturer's instructions. cDNA (1 µl) was used in 20 µl of PCR reaction. A virion-sense 244 RNA standard was made by subcloning PCR products of full length 244 RNA in pGEMT-easy vector (Promega). RNA was transcribed using the T7 RNA polymerase (MEGAscript, Ambion), the mix digested with DNase I, and RNA purified by electro-elution. After ethanol precipitation, RNA was resuspended into RNase-free water and quantitated using a Nanodrop 1000 (Thermoscientific, Wilmington, DE). Standard curves were generated by performing 10-fold serial dilutions of known RNA copy numbers with each dilution assayed in duplicate. The reaction was conducted at 50°C for 2 min, 95°C for 10 min, then 40 cycles of 94°C for 15 sec followed by 60°C for 1 min.

### Infectivity assay

Nasal washes from each ferret were titrated for A/Cal infectivity in a focus-forming assay using MDCK cells in 96-well plates in triplicate. After infection cells were incubated at 33°C for 18 hours, fixed overnight at 4°C with 1∶1 methanol: acetone, and blocked with 5% w/v milk powder in PBS. Virus-positive cells were detected using a mouse monoclonal antibody (9G8, Abcam) that recognises the NP protein of influenza A viruses, and a goat anti-mouse IgG-alkaline phosphatase conjugate (Sigma), both in buffered saline containing 0.1% v/v Tween, and finally incubated with an alkaline phosphatase substrate (NBT/BCIP in TMN buffer; Sigma). At least 50 stained cells (foci) at an appropriate dilution were counted in each of three wells and averaged to give a titre in focus-forming units (FFU) per ferret. Assays carried out on different days were normalized to a standard A/Cal virus preparation. Variation in the standard was less than 4-fold.

### Haemagglutination-inhibition (HI) assay

Before assay, sera were treated with receptor destroying enzyme (RDE II (SEIKEN), Cosmos Biological) overnight at 37°C to remove non-specific inhibitors of haemagglutination and then incubated at 56°C for 30 min to destroy the enzyme. Serial 2-fold dilutions of serum were incubated with 4 HAU of A/Cal for 1 hour at ambient temperature before adding chicken red blood cells (VLA, Weybridge). The HI titre is the dilution of serum that causes 50% inhibition of agglutination, and is interpolated between full agglutination and no agglutination [56].

## Supporting Information

Figure S1 **244 DI virus (244) RNA in nasal washes.** Ferrets were infected with A/Cal on day 0 and treated with 244 DI virus or inactivated 244 DI virus as indicated. Levels of 244 DI RNA were determined by quantitative RT-PCR. Each point represents an individual animal. The horizontal line is the geometric mean for each day, and the dotted line shows the limit of detection.
(TIF)

Figure S2 **Rectal temperatures in ferrets that were rechallenged with A/Cal 21 days after initial infection.** Prior to challenge with A/Cal ferrets were treated with A/Cal+300 µg 244 DI virus (▪); A/Cal+300 µg inactivated 244 DI virus (▴); saline only, and was being infected for the first time (l). Animals were anaesthetised and rectal temperatures taken prior to any other procedure. The mean changes in temperatures of each group (n = 5) are expressed relative to the average temperature of the group immediately prior to the challenge infection on day 0. The fever peak of the virus control occurred on day 2 and differed significantly by a one tailed unpaired t-test from the DI virus-treated group (*, p≤0.03).
(TIF)

Figure S3 **Weight changes in ferret groups rechallenged with A/California/04/09 (H1N1) 21 days after initial infection (day 0 on the graph).** For other information see Figure S2. Mean changes in weight of each group (n = 5) are compared with a baseline established before infection. Animals were anaesthetised and rectal temperatures taken prior to any other procedure. Weights of the group originally treated with DI virus were significantly different by a one tailed unpaired t-test from the group treated with inactivated DI virus: *, p≤0.01; **, p≤0.0009.
(TIF)

Table S1 **Summary of the reduction of respiratory disease (sneezing and nasal discharge) in infected ferrets treated with 244 DI virus or inactivated 244 DI virus.**
(DOCX)

Table S2 **Summary of nasal wash infectivity and 244 DI RNA for ferrets infected with A/Cal and treated with inactivated or active 244 DI virus.**
(DOCX)

Table S3 **Summary of clinical observations in the 7 days following rechallenge of ferrets with A/Cal at 21 days after they were first inoculated.** The accumulated number of single positive events recorded is shown for each group. There were 14 observation periods and 5 ferrets per group, thus there was a total of 70 ferret observations.
(DOCX)
