# Supplementary material for: Cloned Defective Interfering Influenza Virus Protects Ferrets from Pandemic 2009 Influenza A Virus and Allows Protective Immunity to Be Established
Source: PLoS One. 2012 Dec 12;7(12):e49394. doi: 10.1371/journal.pone.0049394 (PMC3521014; doi:10.1371/journal.pone.0049394)
Supplement: Table S1 — Summary of the reduction of respiratory disease (sneezing and nasal discharge) in infected ferrets treated with 244 DI virus or inactivated 244 DI virus. (DOCX) [file pone.0049394.s004.docx]

**Table S1.**

| **Parameter** | **Times of**  **observation^a^** | **P values comparing infected ferrets treated with different amounts of 244 DI virus or inactivated 244 DI virus** | |
| --- | --- | --- | --- |
|  |  | **300 μg** | **30 μg** |
| Sneezing | a.m. + p.m. | 0.048 ^b^ | 0.32 |
| Nasal discharge | a.m. + p.m. | 0.008 ^b^ | 0.009 ^b^ |
| Sneezing + nasal discharge | a.m. + p.m. | 0.006 ^b^ | 0.17 |

^a^ Ferrets were observed twice daily

^b^ p values (one tailed Mann-Whitney U test) showing a significant reduction in clinical signs in 244 DI virus-treated infected ferrets compared with infected ferrets treated with inactivated 244 DI virus.
