# Supplementary material for: Cloned Defective Interfering Influenza Virus Protects Ferrets from Pandemic 2009 Influenza A Virus and Allows Protective Immunity to Be Established
Source: PLoS One. 2012 Dec 12;7(12):e49394. doi: 10.1371/journal.pone.0049394 (PMC3521014; doi:10.1371/journal.pone.0049394)
Supplement: Table S2 — Summary of nasal wash infectivity and 244 DI RNA for ferrets infected with A/Cal and treated with inactivated or active 244 DI virus. (DOCX) [file pone.0049394.s005.docx]

**Table S2.**

| **Day on which:** | **Infectivity in ferrets**  **treated with:** | | | **244 DI RNA in ferrets**  **treated with:** | |
| --- | --- | --- | --- | --- | --- |
|  | **300 μg**  **iDI virus^a^** | **30 μg**  **DI virus** | **300 μg**  **DI virus** | **30 μg**  **DI virus** | **300 μg**  **DI virus** |
| **Significant amounts of**  **infectivity or DI RNA appeared** | 2 ^b^ | 2 | 2 | 2 | 2 |
| **Peak value occurred** | 2 | 3 | 3-5 | 2-3 | 3 |
| **Clearance took place** | 8 | 8 | 8 | 10 | 10 |

^a^ iDI, inactivated 244 DI virus; DI, active 244 DI virus

^b^ Day(s) on which event occurred.
