# Supplementary material for: Cloned Defective Interfering Influenza Virus Protects Ferrets from Pandemic 2009 Influenza A Virus and Allows Protective Immunity to Be Established
Source: PLoS One. 2012 Dec 12;7(12):e49394. doi: 10.1371/journal.pone.0049394 (PMC3521014; doi:10.1371/journal.pone.0049394)
Supplement: Table S3 — Summary of clinical observations in the 7 days following rechallenge of ferrets with A/Cal at 21 days after they were first inoculated. The accumulated number of single positive events recorded is shown for each group. There were 14 observation periods and 5 ferrets per group, thus there was a total of 70 ferret observations. (DOCX) [file pone.0049394.s006.docx]

**Table S3.**

| **Treatment on day 0** |  | Active  244 DI virus (300 μg ) | Inactivated 244 DI virus (300 μg) | Saline | Saline |
| --- | --- | --- | --- | --- | --- |
| **Challenge on day 0 with A/Cal**  **(10^2^ IU /ferret)** |  | + | + | + | - |
| **Rechallenge on day 21 with A/Cal**  **(10^6^ IU /ferret)** |  | + | + | + | + |
| **Parameters of infection on rechallenge** | **Sneezing** | 1^a^ | 1 | 2 | 26 |
|  | **Nasal discharge** | 0 | 0 | 0 | 9 |
|  | **Activity loss** | 0 | 0 | 0 | 25 |
|  | **Appetite loss** | 0 | 0 | 0 | 15 |

+ or -, indicates that the indicated procedure was, or was not, carried out.

^a^ all parameters were recorded twice daily (a.m. + p.m.) and are shown summed here. For example, ‘1’ denotes a single ferret recorded as sneezing once during the whole observation period for that test group. The accumulated number of single positive events recorded is shown for each group. There were 14 observation periods and 5 ferrets per group, thus there was a total of 70 ferret observations.
